# Elevated few-shot network intrusion detection via self-attention mechanisms and iterative refinement

**DOI:** 10.1371/journal.pone.0317713

**Published:** 2025-01-16

**Authors:** Congyuan Xu, Yong Zhan, Guanghui Chen, Zhiqiang Wang, Siqing Liu, Weichen Hu

**Affiliations:** 1 College of Information Science and Engineering, Jiaxing University, Jiaxing, Zhejiang, China; 2 School of Information Science and Technology, Zhejiang Sci-Tech University, Hangzhou, Zhejiang, China; 3 School of Electrical and Information Engineering, Tianjin University, Tianjin, China; Khalifa University, UNITED ARAB EMIRATES

## Abstract

The network intrusion detection system (NIDS) plays a critical role in maintaining network security. However, traditional NIDS relies on a large volume of samples for training, which exhibits insufficient adaptability in rapidly changing network environments and complex attack methods, especially when facing novel and rare attacks. As attack strategies evolve, there is often a lack of sufficient samples to train models, making it difficult for traditional methods to respond quickly and effectively to new threats. Although existing few-shot network intrusion detection systems have begun to address sample scarcity, these systems often fail to effectively capture long-range dependencies within the network environment due to limited observational scope. To overcome these challenges, this paper proposes a novel elevated few-shot network intrusion detection method based on self-attention mechanisms and iterative refinement. This approach leverages the advantages of self-attention to effectively extract key features from network traffic and capture long-range dependencies. Additionally, the introduction of positional encoding ensures the temporal sequence of traffic is preserved during processing, enhancing the model’s ability to capture temporal dynamics. By combining multiple update strategies in meta-learning, the model is initially trained on a general foundation during the training phase, followed by fine-tuning with few-shot data during the testing phase, significantly reducing sample dependency while improving the model’s adaptability and prediction accuracy. Experimental results indicate that this method achieved detection rates of 99.90% and 98.23% on the CICIDS2017 and CICIDS2018 datasets, respectively, using only 10 samples.

## 1 Introduction

Cybersecurity occupies a crucial position in the modern information technology landscape, particularly with the development of NIDS that help protect information systems from malicious traffic attacks. Traditional intrusion detection systems primarily utilize machine learning techniques, such as Support Vector Machines (SVM) [1] and decision trees [2]. While these techniques perform well with ample data, they are heavily reliant on statistical traffic features, making the selection of appropriate statistical features critical to performance. This process is not only time-consuming and labor-intensive but also struggles to adapt to emerging complex threats.

To overcome these limitations, researchers have begun exploring the application of deep learning techniques in intrusion detection, particularly leveraging Convolutional Neural Networks (CNNs), Recurrent Neural Networks (RNNs), and Long Short-Term Memory networks (LSTMs) to automatically extract features from network traffic [3–5], rather than relying on statistical features. Thanks to their advantages in handling large-scale data and feature learning, these deep neural networks have gradually become dominant in the field of network traffic intrusion detection [6].

Nevertheless, as network threats continue to evolve, there is often insufficient time to collect enough malicious samples for training, and traditional deep learning methods significantly underperform when faced with novel and rare threats. These methods typically struggle to accurately identify or adapt to unseen attack patterns [7], particularly in few-shot or zero-day attack scenarios [8]. Consequently, these models face challenges due to insufficient training samples, leading to decreased adaptability and generalization capabilities. Effectively learning from limited samples and swiftly adapting to new threats has become a focal point in the development of NIDS. In recent years, few-shot learning has emerged as a research hotspot in deep learning [9]. Techniques such as meta-learning, metric learning, and data augmentation have been proposed to address the poor performance of deep learning methods in few-shot environments, and these methods are equally applicable in the field of cybersecurity [10]. By simulating few-shot scenarios to optimize the learning process of models, their ability to recognize novel and rare threats has been enhanced, with relevant algorithms showing preliminary success in the cybersecurity domain.

Considering the limitations of existing intrusion detection models regarding data requirements and global information capture, this paper proposes a novel few-shot network intrusion detection method. Leveraging the advantages of self-attention mechanisms [11], the method effectively extracts long-range dependencies in key features. Utilizing a multi-update meta-learning framework, the model parameters are progressively adjusted and optimized, significantly enhancing the model’s learning capability and adaptability to new threats. The proposed model, based on self-attention mechanisms, consists of three main components: an embedding layer, an encoding layer, and a classifier. The embedding layer first converts input data into vector representations in high-dimensional space, capturing the complex relationships among input features more effectively. The encoding layer then processes these vectors through the self-attention mechanism, allowing the model to strengthen representations of the most critical information while suppressing irrelevant or noisy data. Finally, the classifier makes decisions based on the output from the encoding layer, achieving precise classification results. During the training and testing phases, an innovative multi-update strategy is employed, dividing the model’s update process into two main loops: the inner loop and the outer loop. The inner loop quickly adapts to the specific samples of each task through a few gradient updates, while the outer loop optimizes the model’s generalization ability across multiple tasks, ensuring that the model performs well on new, unseen data. This design enables the model to achieve efficient learning for specific tasks while greatly enhancing its stability and generalization across various application scenarios.

This method not only effectively overcomes the limitations of traditional CNNs, RNNs, or LSTMs [12] in handling network security issues but also adapts well to few-shot environments, allowing the model to quickly recognize and respond to new network threats. The main contributions of this paper are as follows:

1. We propose an elevated few-shot network intrusion detection method via self-attention mechanisms and iterative refinement strategies, combining self-attention to capture long-range dependencies and iterative refinement strategies to enhance the model’s performance in few-shot environments.
2. We conduct experiments on the CICIDS2017 and CICIDS2018 datasets, based on real network traffic, achieving detection rates of 99.90% and 98.23%, respectively, with only 10 samples.
3. We compare our proposed method with other related works, demonstrating that our approach achieves higher accuracy with fewer samples compared to traditional deep learning methods, and achieves higher accuracy than existing few-shot network intrusion detection methods.

The following sections of this paper are structured as follows: Section 2 reviews related works, Section 3 systematically describes the proposed detection method, Section 4 evaluates the experiments, Section 5 discusses the findings and compares them with similar studies, Section 6 outlines the study’s limitations, and finally, Section 7 concludes the paper and provides an outlook for future research.

## 2 Related work

### 2.1 Network intrusion detection

Network intrusion detection is a critical issue in the field of network security, aimed at identifying malicious activities within a network to safeguard systems and data. With the emergence and development of deep learning, methods combining deep learning with network intrusion detection have gradually evolved. Shone et al. [13] proposed a novel classification model that integrates deep learning and machine learning, specifically a stacked non-symmetric deep autoencoder (NDAE) combined with a random forest (RF) classification algorithm. This approach leverages the stacking capabilities of NDAEs along with the accuracy and speed of RF. Gurung et al. [14] designed a system using sparse autoencoders, a deep learning method, for intrusion detection. This system not only learns but also adapts to previously undefined patterns. Sparse autoencoders have been used for unsupervised feature learning, and the data is subsequently classified using a logistic classifier. Additionally, R et al. [3] introduced some techniques from the field of computer vision, utilizing CNNs to extract features from network traffic. The authors primarily employed supervised learning methods such as multilayer perceptrons (MLP), CNNs, CNN-Recurrent Neural Network (CNN-RNN), CNN-Long Short-Term Memory network (CNN-LSTM), and CNN-Gated Recurrent Unit (CNN-GRU), which exhibit stronger feature extraction capabilities compared to traditional machine learning methods. Liu et al. [15] used CNNs as the foundational network model and applied Fourier transforms to convert network traffic into images. Since the Fourier transform operates on numerical sequences and network traffic data is not purely numerical, the authors first converted the traffic into numerical sequences, which were then directly subjected to Fourier transformation.

Recent advancements have been made in the integration of deep learning and network intrusion detection. Wang et al. [16] proposed an intrusion detection model using ResNet, Transformer and BiLSTM (Res-TranBiLSTM) that takes into account both the spatial and temporal features of network traffic. They used the Synthetic Minority Over-sampling Technique (SMOTE) combined with the Edited Nearest Neighbor (ENN) method to alleviate the degree of data imbalance. Ullah et al. [17] introduced a transformer neural network-based intrusion detection system (TNN-IDS) designed explicitly for IoT networks supporting MQTT, leveraging the parallel processing capabilities of the transformer neural network to accelerate the learning process and improve malicious attack detection. Kilincer et al. [18] emphasized the importance of building a comprehensive intrusion detection framework. By utilizing boosting algorithms, such as AdaBoost and gradient boosting, they improved the detection accuracy of various network attacks in heterogeneous network environments. Long et al. [19] proposed a novel NIDS algorithm based on the transformer model, integrating the fundamental aspects of network intrusion detection with the inherent complex attention mechanism of the transformer model, and thoroughly examining the relationship between input features and different types of intrusions. Ullah et al. [20] proposed a transformer-based transfer learning method for imbalanced network intrusion detection systems (IDS-INT). IDS-INT uses a transformer-based transfer learning method to learn the network feature representations and interactions in imbalanced data. Simultaneously, a hybrid method of the CNN-LSTM model was developed to detect different types of attacks from deep features. Reka et al. [21] designed a clustering-gradient-based centrality coating optimization algorithm for multi-attack intrusion identification. This method maximizes the attack identification rate of the intrusion detection system without affecting its effectiveness. It utilizes the proposed Coati optimization algorithm and a constitutive artificial neural network (CANN) to select cluster heads and reduce cluster creation in network activity to evaluate the proposed method. Altaf et al. [22] designed a framework called the Node Edge-Graph Convolutional Network (NE-GConv), which enhances the detection capability of the model by utilizing both node and edge features simultaneously. In addition, a novel undersampling method based on the KNN principle and the borderline-SMOTE oversampling method were introduced for mixed data sampling to balance the dataset while addressing the issue of low detection accuracy in overlapping data classes. Manocchio et al. [23] introduced the flowtransformer framework, which is a new approach for implementing a NIDS based on a transformer. This method leverages the advantages of the transformer model in recognizing a network’s long-term behavior and characteristics.

### 2.2 Self-attention mechanism

Self-attention mechanism has achieved significant success in natural language processing. It has been widely applied in computer vision, speech processing, time-series analysis, and other fields. In network traffic analysis, we can utilize high-performance feature extraction and the ability to capture long-distance dependencies offered by a self-attention mechanism. Researchers have begun to explore the application of self-attention mechanisms to network traffic feature learning and threat detection. Transformers, as representatives of self-attention mechanisms, have gradually become a research focus. Huang et al. Han et al. [24] proposed a new transformer-based network in the style of U-Net for repair tasks, called the Sparse Self-Attention Transformer (spa-former). This method employs a new channel attention approximation algorithm that reduces attention computation to linear complexity and effectively excludes irrelevant features by generating sparse attention maps. Han et al. [25] proposed a new intrusion detection model based on n-gram frequency and a time-aware transformer that considers the features of various components of the original network traffic and can hierarchically learn the packet-level and session-level features of the original network traffic. Zhang et al. [26] introduced a deep-learning-based intrusion detection method that integrates the Transformer and LSTM modules with the intrusion detection model to automatically detect network intrusions. They used a Transformer and LSTM to capture the sequential information of traffic data, which is beneficial for distinguishing between abnormal and normal data. Similarly, Li et al. [27] proposed a network intrusion detection method based on a transformer, and designed a dual-encoder transformer that uses weighted sums and residual connections to integrate packet-level and flow-level network traffic features. Expanding the application of Transformers, Zhang et al. [28] introduced an intrusion detection model that combines convolutional neural networks and a transformer, which can capture not only the global relevance between packets but also the local relevance of intrusion behaviors. Barut et al. [29] proposed a malware traffic classification system called the Residual One-Dimensional Image Transformer (R1DIT), which uses raw data transformation and attention-based modules for classification. This system focuses on leveraging knowledge from the network domain by parsing IP, HTTP, DNS, and headers of unencrypted TLS records as byte sequence inputs. Moreover, Fang et al. [30] proposed a Packeted Window Transformer model (PWT) based on learnable embedding features, which learns the embedding features of network traffic by blocking windows of packets of specific sizes, thereby ultimately determining abnormalities in network traffic. Rendón-Segador et al. [31] drew an analogy between network traffic features and the NLS format of natural language sentences. They proposed the application of an attention mechanism for classifying network flows, focusing on the interrelationships between features. By utilizing multi-head self-attention layers, they aimed to enhance the parsing and classification of features. Zhou et al. [32] proposed a novel self-supervised attention generative adversarial image painting method based on a transformer, utilizing the self-attention strategy of the transformer to capture global semantic data and established a self-supervised attention module within the transformer to overcome the limitations of convolutional operations. These methods require a large number of samples for model training, making it difficult to deal with new types of network intrusions with smaller samples.

### 2.3 Few-shot network intrusion

Although deep learning methods combined with network intrusion detection possess strong feature extraction capabilities and perform well in most environments, they rely on large amounts of data. In reality, newly emerging network traffic data to be classified are often scarce, representing few-shot conditions. Therefore, effectively detecting intrusion traffic under few-shot conditions remains an urgent problem to be addressed. To tackle this challenge, the field of network intrusion detection is leveraging mature few-shot methods from other domains, such as meta-learning, transfer learning, and data augmentation and generation techniques. For instance, Xu et al. [33] designed FC-Net, a deep neural network that learns feature maps from raw traffic data to classify new traffic types with few samples. Similarly, Lu et al. [9] introduced a MAML-based method for IoT environments, enabling rapid adaptation to new intrusion types using a Convolutional Neural Network (CNN). Mahdavi et al. [34] developed the ITL-IDS framework, which employs transfer learning and incremental clustering to efficiently detect intrusions with limited data. Additionally, He et al. [35] combined Generative Adversarial Networks (GAN) with meta-learning to enhance few-shot attack sample augmentation and improve detection performance. Focusing on IoT, Yan et al. [36] proposed the GDE Model, which transforms one-dimensional traffic data into two-dimensional images, achieving high accuracy and fast inference. In another approach, Sun [37] utilized attention mechanisms and prototype capsule networks to enhance feature extraction and classification for few-shot intrusion detection. Yan et al. [38] presented a model that integrates an improved GAN and multi-head attention mechanisms to boost detection accuracy and real-time performance. Meanwhile, Tong et al. [39] developed IResTAE2A, a method combining self-supervised and supervised learning with efficient channel attention to better extract hidden information from normal traffic without labeled data. Wang et al. [40] introduced BT-TPF, a lightweight model that uses a Siamese network for feature reduction and knowledge distillation from a Vision Transformer to a smaller Poolformer model, maintaining high accuracy with fewer parameters. Overall, these studies highlight significant progress in few-shot network intrusion detection through the integration of meta-learning, transfer learning, and attention mechanisms. Building on these advancements, this paper proposes a novel few-shot network intrusion detection method based on self-attention and multiple updates, leveraging the self-attention mechanism’s ability to capture long-distance dependencies and facilitate rapid model optimization.

## 3 Method

The few-shot intrusion detection method that we proposed, utilizes the raw network traffic provided by the Canadian Institute for Cybersecurity (CIC) [41]. Through the self-attention mechanism, the model learns the feature relevance of the original network traffic and applies the principles of meta-learning. Specifically, the model is initially trained on existing tasks to obtain a good set of initial parameters, and then trained on new tasks, enabling the model to quickly learn and adapt to emerging network intrusion scenarios. This iterative refinement process, combined with self-attention, enhances the model’s capability to perform effective intrusion detection even under few-shot conditions.

### 3.1 Data preprocessing

The method uses the raw network traffic provided by the CIC, including CSV files and raw traffic PCAP packets. The CSV files contain the source IP, source port, destination IP, destination port, and classification labels of the data flows. Labels corresponding to each data flow in the PCAP packets are attached based on the CSV files, and original IPs are replaced with randomly generated IPs. The data is further processed using the data preprocessing method of Xu et al. [33]. Specifically, original IPs are replaced with randomly generated IPs for anonymization, then the first 256 bytes of the first 16 packets of each data flow are extracted. The extracted data is divided into training and testing sets by task, and further divided into support sets and query sets based on the concept of meta-learning.

### 3.2 Gradient adaptive attention model

The self-attention mechanism can extract features with high relevance from global information by calculating the degree of correlation between network traffic, while information with lower relevance is weakened. Unlike traditional machine learning, this model does not learn the features of a specific target sample but is based on the task to learn how to acquire the capability to learn new tasks. Specifically, data is divided by task. In the training phase, existing tasks are learned first, and then on this basis, tasks that have never been seen before are learned, increasing their generalization ability. Finally, through multiple training sessions, a model with a good set of initial parameters is obtained, which can quickly adapt to new tasks in a few-shot manner.

**Fig 1:**
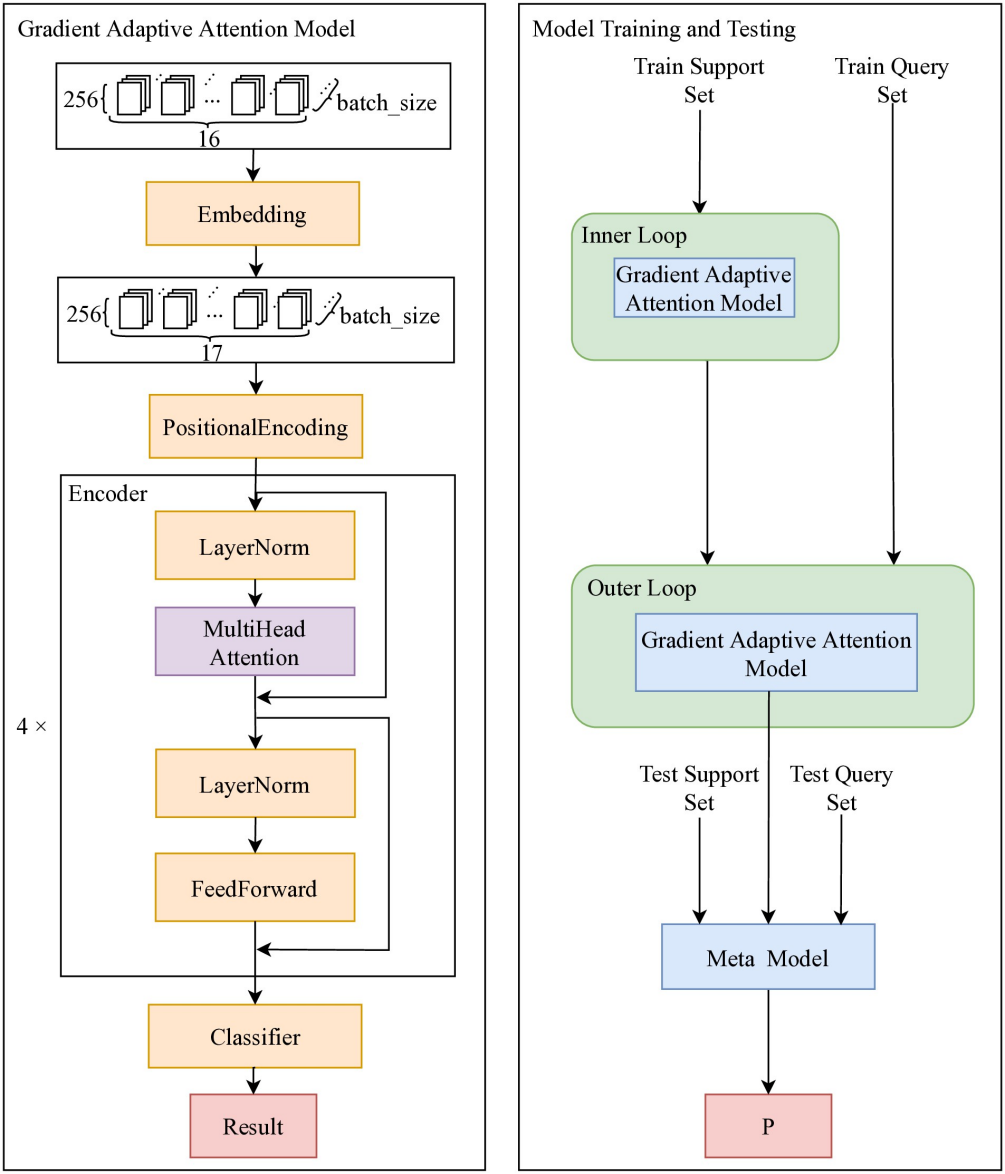
The training, testing and the internal structure of the model.

Fig 1 shows the framework of the model, and the right half shows its core structure. After preprocessing the data, each sample data *x*_1_*x*_2_…*x*_16_ in the input task is vector-embedded in the learnable vector embedding layer to be transformed into *x*_0_*x*_1_…*x*_16_. This implies that a learnable embedding vector is inserted at the beginning of each data stream. This embedding vector can automatically learn the features of the original traffic through a self-attention mechanism during the model training phase and serve as the basis for classifier classification.

The sequential relationship between each flow is also significant in the network. To represent the sequence among the flows, positional encoding using sine and cosine functions was applied in the position embedding layer to the input vectors *x*_1_*x*_2_…*x*_16_ to add positional information. The input data become $x_{0}^{\prime }{\ldots x}_{16}^{\prime }$ mathematical representation is shown in Eq (1).

$$\begin{array}{c} \left\{ \begin{array}{cc} & P_{\left(p,2i\right)}=\text{sin}(\frac{p}{10000^{\frac{2i}{d}}})\\ & P_{\left(p,2i+1\right)}=\text{cos}(\frac{p}{10000^{\frac{2i}{d}}}) \end{array}\right. \end{array}$$ (1)

where *p* represents *p*^*th*^ the flow of the 16 flows, *d* represents the dimensions of each flow (256 bytes), and *i* represents the *i*^*th*^ byte of a single flow. The term $10000^{\frac{2i}{d}}$ was used to allow the model to better capture positional information when processing flows. It is important to note that current common encoding methods also include Learned Positional Encoding [11] and Relative Positional Encoding [42], among others. In contrast, sinusoidal positional encoding does not require additional parameters and is straightforward to implement, thereby maintaining the model’s simplicity and training efficiency.

The training process of the model is shown in the left half of Fig 1, where the internal detailed structure of the gradient adaptive attention model is on the right side. During the entire training phase, the first multi-head self-attention encoder is trained in the inner loop, then its weight parameters are passed to the multi-head self-attention encoder in the outer loop for training, and finally, the model is fine-tuned on the test set to obtain the final target detection model.

In the encoding layer, to make the model converge quickly, the data is first normalized and then fed into the multi-head self-attention module. The multi-head self-attention module splits a sample and performs multiple self-attention operations in different spaces in parallel. This module not only learns the features of the traffic in different spaces but also reduces the training time of the model.

The self-attention operation generates three vectors, *Q*, *K*, *V* for each input vector through a linear transformation. It calculates the relevance between every pair of input vectors using the *Q* and *K* vectors, performs layer normalization, and finally conducts a residual connection with the *V* vector, enhancing the feature transmission capability. The self-attention mechanism can effectively learn the relational features of traffic. The specific operation process is shown in Eq (2).

$$\begin{array}{c} \text{Attention}\left(Q,K,V\right)=\text{softmax}(\frac{QK^{T}}{\sqrt{d_{k}}})V \end{array}$$ (2)

where *Q* represents the specified traffic information, *K* represents the traffic to be matched, and the relevance of each traffic flow is obtained through dot-product operations. *V* represents the original information of the specified traffic.

Multi-head self-attention calculates self-attention in different subspaces independently and in parallel by dividing the input vector into different “heads.” Each head calculates self-attention, and after completion, all results are concatenated to form the self-attention matrix and multiplied by a weight matrix *W*_0_ to obtain the final self-attention. Different heads focus on various types of information, which can effectively prevent the problem of attention from being too concentrated. Specific details are given in Eq (3).

$$\begin{array}{c} \text{MultiAttention}=W_{0}{\left[a_{1}\ldots \right]}^{T} \end{array}$$ (3)

where *a*_1_ represents the self-attention matrix of the first head, and *W*_0_ is the corresponding weight matrix generated by a linear transformation. After passing through the multi-head self-attention module, it undergoes layer normalization and is fed into a feed-forward neural network. Finally, it undergoes residual connection and output, learning more features in the traffic.

The output of the multiple encoding layers is fed into the classification layer, which uses normalization, linear transformation, and a sigmoid function to map features to the prediction probabilities of the two categories. The results were classified as normal and attack traffic based on the predicted probabilities. Subsequently, the Adam gradient descent algorithm and backpropagation are used to update the weight parameters of the model.

Based on the training process shown in Fig 1, a specific training algorithm can be designed, as shown in Algorithm 1, with the following key processes:

**Algorithm 1** Iterative Refinement Intrusion Detection Training Algorithm

**Input**: *τ*_*n*_: Distribution of intrusion detection tasks

**Output**: *α*,*β*: Learning rate

**Require**: update:Number of updates

1 Random initialization of *θ*

2 **for** *all epoch* **do**

3  Randomly select a batch of tasks *τ*_*i*_ ∈ *τ*_*n*_

4  **for** *all τ*_*i*_ **do**

5   Support set *D*_*s*_ containing K samples from $\tau_{i}=\{X_{s}^{\left(j\right)},Y_{s}^{\left(j\right)}\}$

6   Compute the gradient $L_{\tau_{i}}\left(f_{\theta }\right)$ based on K samples and the loss function $L_{\tau_{i}}$

7   Compute the adaptive parameter for gradient descent:

8   *θ*′ = *θ* − *α*∇_*θ*_ $L_{\tau_{i}}\left(f_{\theta }\right)$

9   Update self-attention weights based on adaptive parameters

10   $W_{attention}^{\prime }=g\left(\theta^{\prime }\right)W_{attention}$

11   Query set *D*_*q*_ containing *k*′ samples from $\tau_{i}=\{X_{q}^{\left(j\right)},Y_{q}^{\left(j\right)}\}$

12   Calculate the loss of the model after parameter updates

13   **for** *j = 1 to update* **do**

14    Compute the gradient $L_{\tau_{i}}\left(f_{\theta^{\prime }}\right)$ based on K samples and the loss function $L_{\tau_{i}}$

15    Compute the adaptive parameter for gradient descent:

16    *θ*′ = *θ*′ − *α* $\nabla_{\theta^{\prime }}L_{\tau_{i}}\left(f_{\theta^{\prime }}\right)$

17    Update self-attention weights based on adaptive parameters

18    $W_{attention}^{\prime }=g\left(\theta^{\prime }\right)W_{attention}$

19    Query set *D*_*q*_ containing *k*′ samples from $\tau_{i}=\{X_{q}^{\left(j\right)},Y_{q}^{\left(j\right)}\}$

20    Calculate the loss of the model after parameter updates

21   **end**

22  **end**

23  Compute the gradient based on the total loss of the query set from *τ*_*i*_ and the loss function

24  Update *θ* ← *θ* − *β*∇_*θ*_ $\sum_{\tau_{i}∼\tau_{n}}L_{\tau_{i}}\left(f_{\theta^{\prime }}\right)$

25 **end**

A batch of tasks *τ*_*i*_ is randomly drawn from *τ*_*n*_, including a training support set and a training query set for model training. The model is pre-trained using the training support set, the loss on the support set is calculated using the cross-entropy loss function, and the model’s weight parameters are updated through Adam gradient descent and backpropagation, obtaining the first updated parameters *θ*′. Subsequently, the loss is calculated for the multi-head self-attention encoder with the updated parameters using the query set. The formula for calculating the loss function is given in Eq (4):

$$\begin{array}{c} L=\frac{1}{N}\sum_{i}-[(y_{i}\cdot {\text{log}}_{e}p_{i}+\left(1-y_{i}\right)\cdot {\text{log}}_{e}\left(1-p_{i}\right))] \end{array}$$ (4)

The weight parameters were updated through multiple cycles, repeating steps 5~10 to capture more target task feature information through multiple update cycles and improve the adaptability of the model. Notably, the tasks performed in each cycle differed. Based on the aforementioned weight parameters, the model’s weight parameters were updated using the training query set, aiming to train the model’s ability to learn new tasks, specifically using the loss calculated on the query set to update the weight parameters of the multi-head self-attention encoder for the second time. The initial weight parameters of the multi-head self-attention encoder in the outer loop are the weight parameters of the multi-head self-attention encoder trained in the inner loop. The complete training process can be expressed with Eq (5):

$$\begin{array}{c} p\left(D_{T}^{q}|\theta_{o},D_{T}^{t}\right)=\prod_{\tau \in T}p\left(D_{\tau }^{q}|\theta_{\tau }^{\prime }=\theta_{0}+\alpha \nabla_{\theta_{0}}\;\text{log}\;p\left(D_{\tau }^{S}|\theta_{0}\right)\right) \end{array}$$ (5)

where $p(D_{\tau }^{s}|\theta_{0})$ represents the likelihood probability of the model in the support set $D_{\tau }^{s}$ within the inner loop multi-head self-attention encoder, by continuously updating the parameters *θ*′, updating the posterior of the inner loop multi-head self-attention encoder, and finally making $\theta_{\tau }^{\prime }$ act as the prior of the outer loop multi-head self-attention encoder on the query set $D_{\tau }^{q}$.

## 4 Evaluation

### 4.1 Dataset

To comprehensively evaluate the effectiveness of our proposed few-shot network intrusion detection method, this study selected the widely recognized CICIDS2017 and CICIDS2018 public datasets in the field of cybersecurity. The total volume of the pcap dataset reached 452 GB, including 48.8 GB from CICIDS2017 and 403.2 GB from CICIDS2018. This dataset encompasses a diverse range of attack types, ensuring strong generalizability. As shown in Table 1, these datasets contain a large amount of real intrusion traffic. We selected 1000 samples from each attack type. This can effectively cover the variability and complexity of attack behaviors, helping the model to learn richer and more detailed content, thus improving accuracy and generalization capabilities [43] without causing excessive consumption of computational resources due to large data volumes.

**Table 1:** Attack types distribution in CICIDS2017 and CICIDS2018 datasets.

Attack Type	CICIDS2017 Samples	CICIDS2018 Samples
DoS	N/A	1000
DDoS	1000	1000
Brute Force	1000	1000
Web Attacks	1000	N/A
Port Scan	1000	N/A
Botnet	N/A	1000
Benign	4000	4000

### 4.2 Evaluation metrics

We used commonly accepted evaluation metrics to compare the proposed method with related methods. Five metrics were used: Accuracy, Detection Rate, Precision Rate, F1-Score, and Specificity, as shown in Eq (6). These metrics are calculated based on the confusion matrix components: True Positives (TP), True Negatives (TN), False Positives (FP), and False Negatives (FN).

$$\begin{array}{c} \left\{ \begin{array}{cc} & \text{Accuracy}\;\text{(ACC)}=\frac{\text{TP+TN}}{\text{TP+FP+FN+TN}}\\ & \text{Detection}\;\text{Rate}\;\text{(DR)}=\frac{\text{TP}}{\text{TP+FN}}\\ & \text{Precision}\;\text{Rate}\;\text{(PR)}=\frac{\text{TP}}{\text{TP+FP}}\\ & \text{F1-Score}=\frac{2\times \text{DR}\times \text{PR}}{\text{DR+PR}}\\ & \text{Specificity}=\frac{\text{TN}}{\text{TN+FP}} \end{array}\right. \end{array}$$ (6)

### 4.3 Hyperparameter settings

**Fig 2:**
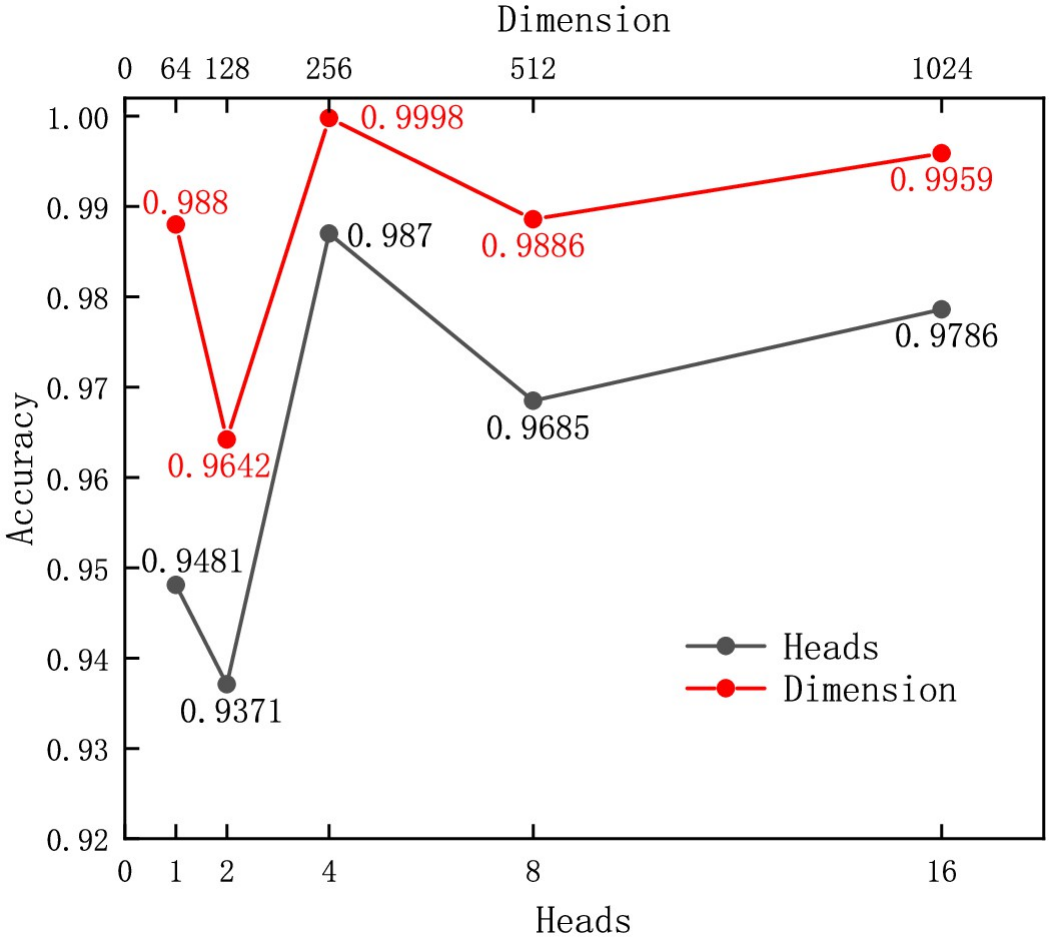
The accuracies of the models in different hyperparameter settings.

This study focused on analyzing the hyperparameters that significantly affect model performance, including the number of attention heads and feed-forward neural network dimensions (FFD). To determine the optimal hyperparameter configuration, we designed a series of experiments to evaluate the changes in model performance by varying the values of these parameters. The accuracy was used as the evaluation metric, and the experimental results are shown in Fig 2, from which we can observe the impact of different hyperparameter configurations on the model accuracy.

Fig 2 shows that increasing the number of attention heads and FFD generally led to an upward trend in accuracy. The best performance was achieved with four layers and the FFD of 256, with accuracies of 99.98% and 98.70%, respectively. Although increasing the number could increase the accuracy further, this would make the training more time-consuming. Thus, considering the balance of various factors, the hyperparameters listed in Table 2 were chosen for subsequent experiments.

**Table 2:** Hyperparameter settings.

Hyperparameter Settings	Value
FFD	256
Attention Heads	4
Learning Rate	2 × 10^−3^
batch size	8

### 4.4 Experimental setup

The software and hardware environment for the experiments in this study were as follows: The CPU used was an Intel(R) Xeon(R) Platinum 8358C CPU @ 2.60GHz, and the GPU was an RTX A5000 (24 GB). The operating system used was Ubuntu 20.04, Python version 3.8, and the deep learning framework was PyTorch 1.10.0. The GPU acceleration library was CUDA 11.3, with a memory size of 128 GB.

To quantify the impact of training rounds and sample size on detection performance, the experiments were divided into three groups: Group A, to explore the impact of training rounds, and Group B, to explore the impact of sample quantity. Additionally, Group C evaluated inference time, parameter size, and the effects of iterative refinement on performance.

**Group A**: To ensure that the model converges properly and delve deeper into the impact of training rounds on performance metrics, we averaged the metrics for all available attack types in CICIDS2017 and CICIDS2018. The accuracies and detection rates were then plotted using trend lines to reflect the overall change trend of the model and show the evolution of the overall model performance. In addition, we evaluated the performance of the method on two datasets using the F1-Score.

**Group B**: To explore the impact of varying sample sizes on model performance, the following experiment was designed with training and testing conducted on the CICIDS2017 and CICIDS2018 datasets, using sample sizes of 1, 3, 5, and 10. These experiments were aimed at simulating a novel attack scenario. Thus, we selected attack types that were previously unseen by the models as the test set. The generalizability of the model was systematically evaluated using multiple datasets and different metrics. Moreover, we explored the dependence of this method on the number of samples compared to traditional methods.

**Group C**: To investigate the model’s inference time and parameter count, and to analyze the impact of the number of iterative refinement cycles on accuracy and runtime, we designed the following experiments. For a fair comparison on the CICIDS2018 dataset, our method utilized 10 samples, whereas other methods employed 100 samples to ensure that the comparative methods could leverage more data to optimize their performance. We examined the effect of varying the number of iterations on model performance. Additionally, we compared our method with several commonly used approaches in terms of parameter count and accuracy, and evaluated the model’s inference time to comprehensively assess the efficiency and practicality of the proposed method.

### 4.5 Detection results

**Fig 3:**
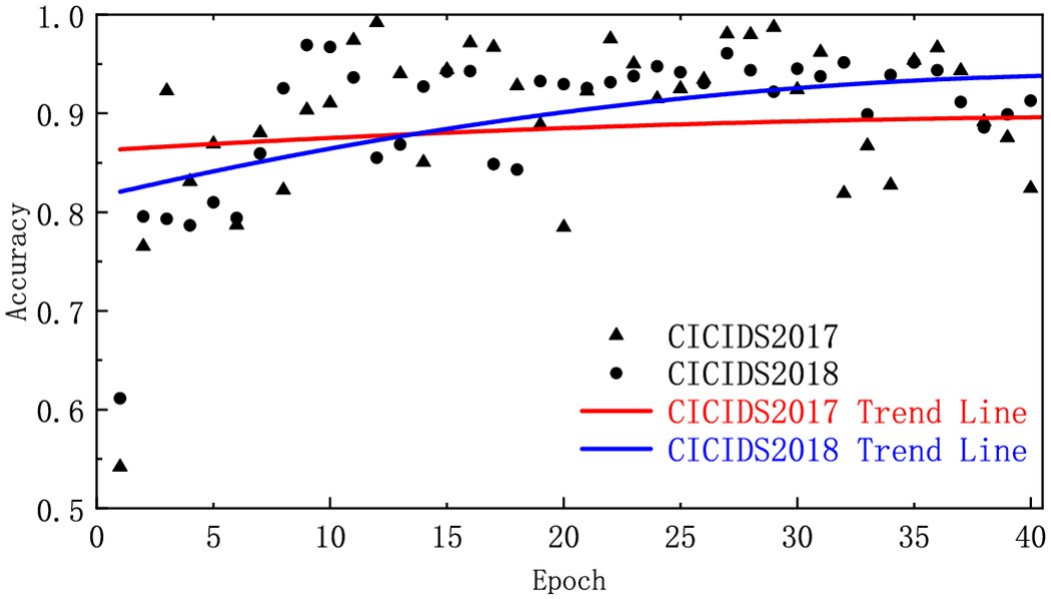
The accuracy in different datasets.

**Fig 4:**
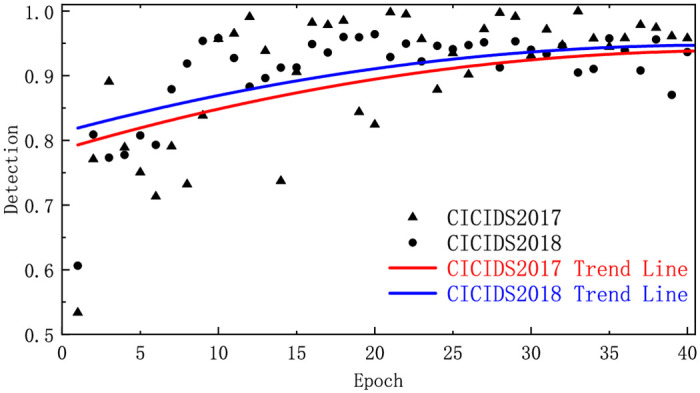
The detection rates in different datasets.

**Group A**: As shown in Figs 3 and 4, within the first 25 epochs, the Accuracy of CICIDS2017 and CICIDS2018 rapidly increased, stabilizing from 25 to 40, with most of the accuracy and detection rates in this range falling between 0.9 and 1.0. This indicates that the model requires only a few training cycles to converge, with subsequent cycles having little impact on performance improvement. As shown in Table 3, the F1-Score metrics can reach 77.03% and 80.03%, respectively, with only two epochs; compared to one epoch, they increase by 35.28% and 31.59%, respectively. With only 10 epochs, they reached 92.72% and 96.69%, respectively, with increases of 19.56% and 20.82%, respectively, compared to two epochs.

**Table 3:** Relationship between F1-Score and epoch.

Epoch	F1-Score	
	CICIDS2017	CICIDS2018
1	53.84%	60.84%
2	77.03%	80.03%
5	85.20%	81.24%
10	92.72%	96.69%

**Fig 5:**
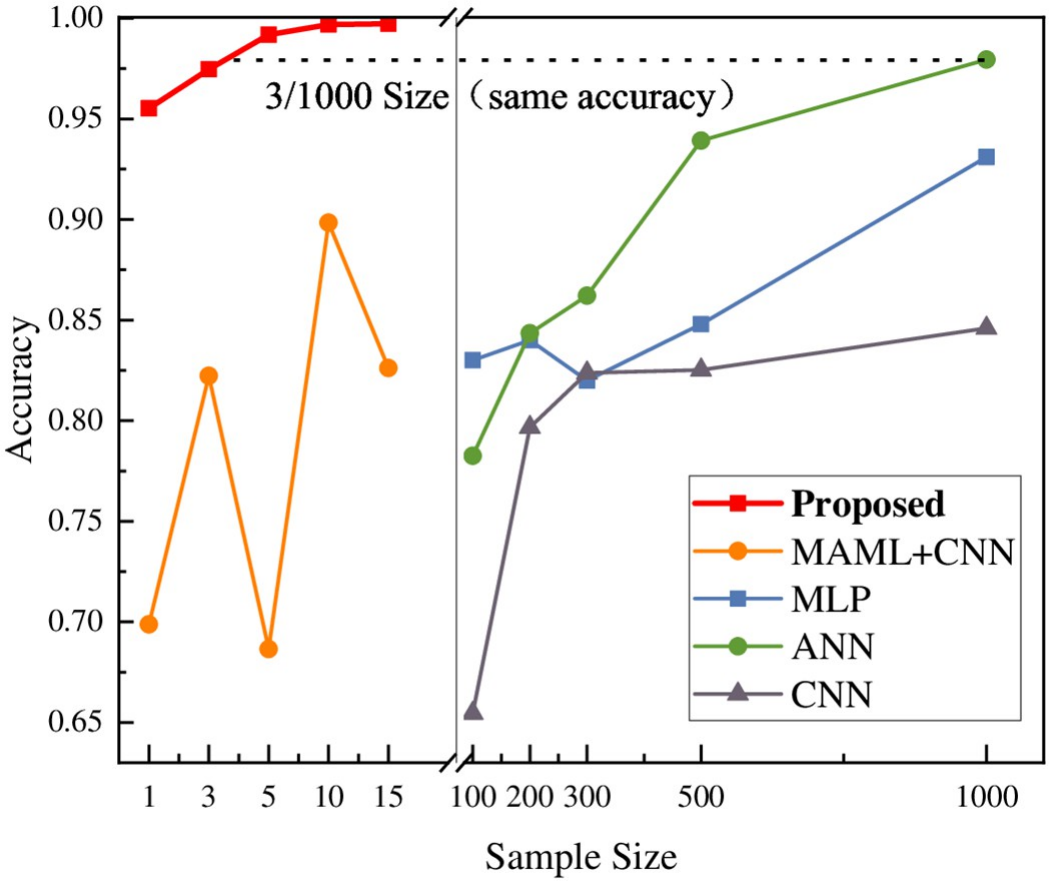
Accuracy of different models in the CICIDS2017 dataset.

**Group B**: As presented in Tables 4 and 5, for K = 1, 3, 5, and 10, the model performed excellently under few-shot scenarios for both the CICIDS2017 and CICIDS2018 datasets. For the CICIDS2017 dataset with only one sample, the accuracy reached 95.52% and the detection rate was 96.74%. Even with a smaller number of samples, the model maintained high detection performance. In the CICIDS2018 dataset, despite the small number of samples, the model achieves high accuracy and detection rates, especially in the cases of K = 5 and K = 10, where the accuracy exceeded 96%. As shown in Fig 5, the left side of the image displays the relationship between the model accuracy and sample size in the few-shot domain. In contrast, the right-hand side displays the relationship for traditional intrusion detection models, such as MLP, ANN, and CNN. Traditional models require a larger number of samples for better experimental results; however, obtaining a sufficient number of samples is difficult for network intrusion detection. The ANN model needs 1000 samples to achieve the same accuracy, whereas the proposed model required only three samples. In the few-shot scenario, we developed the MAML + CNN model and found that it fluctuates more in ordinary few-shot environments. Under the same sample conditions, our proposed model can achieve higher accuracy. This further demonstrates that our model requires fewer samples and exhibits higher stability.

**Table 4:** The results in the CICIDS2017 dataset.

Sample	ACC (%)	DR (%)	PR (%)	Specificity (%)
1	95.52	96.74	94.44	94.31
3	97.46	97.26	97.64	97.66
5	99.18	99.06	99.29	99.29
10	99.69	99.90	99.48	99.48

**Table 5:** The results in the CICIDS2018 dataset.

Sample	ACC (%)	DR (%)	PR (%)	Specificity (%)
1	92.61	89.79	95.27	95.43
3	92.24	92.60	92.02	91.88
5	96.91	95.36	98.43	98.45
10	98.75	98.23	99.26	99.27

**Fig 6:**
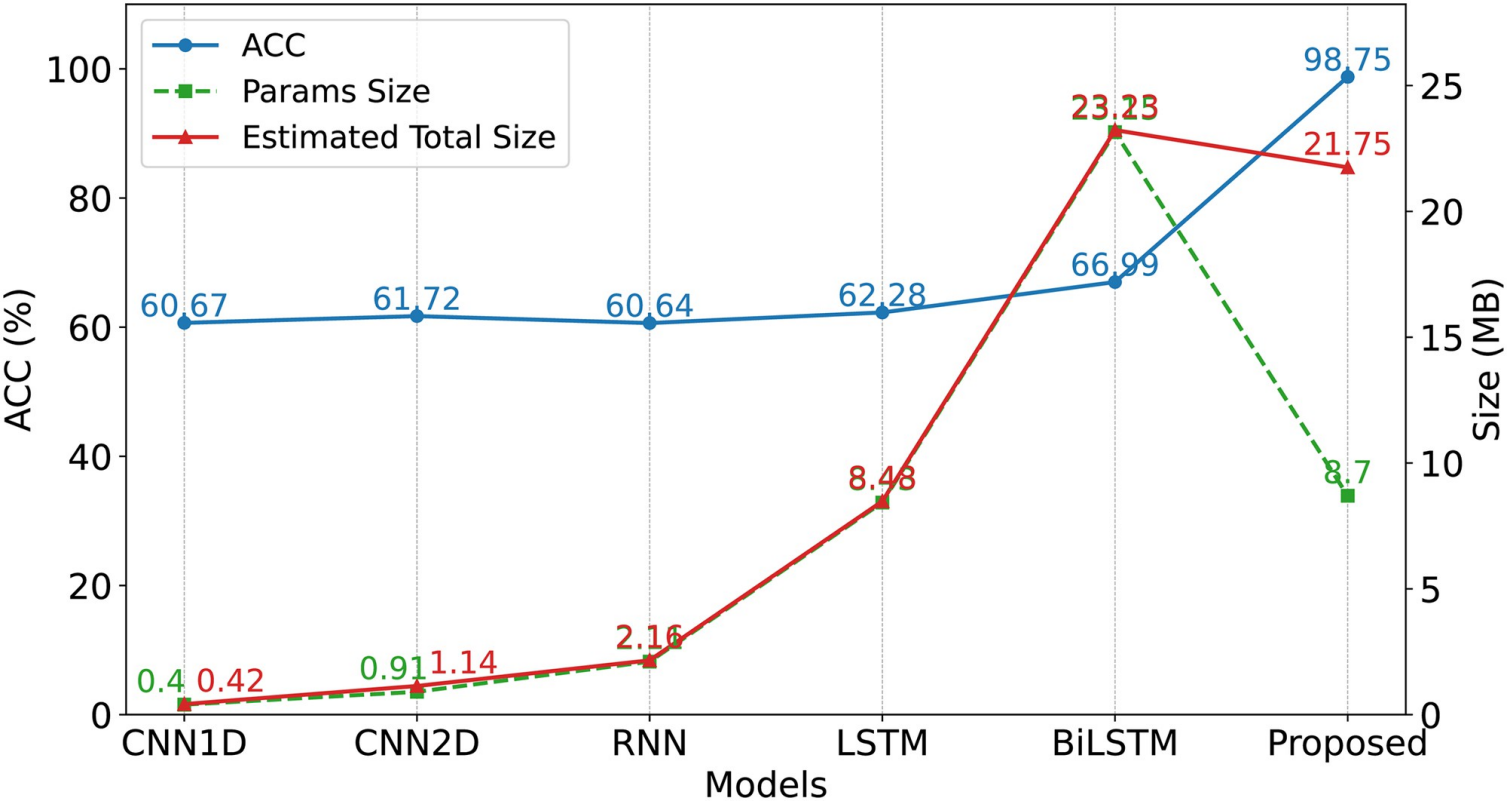
Model accuracy and size comparison.

**Fig 7:**
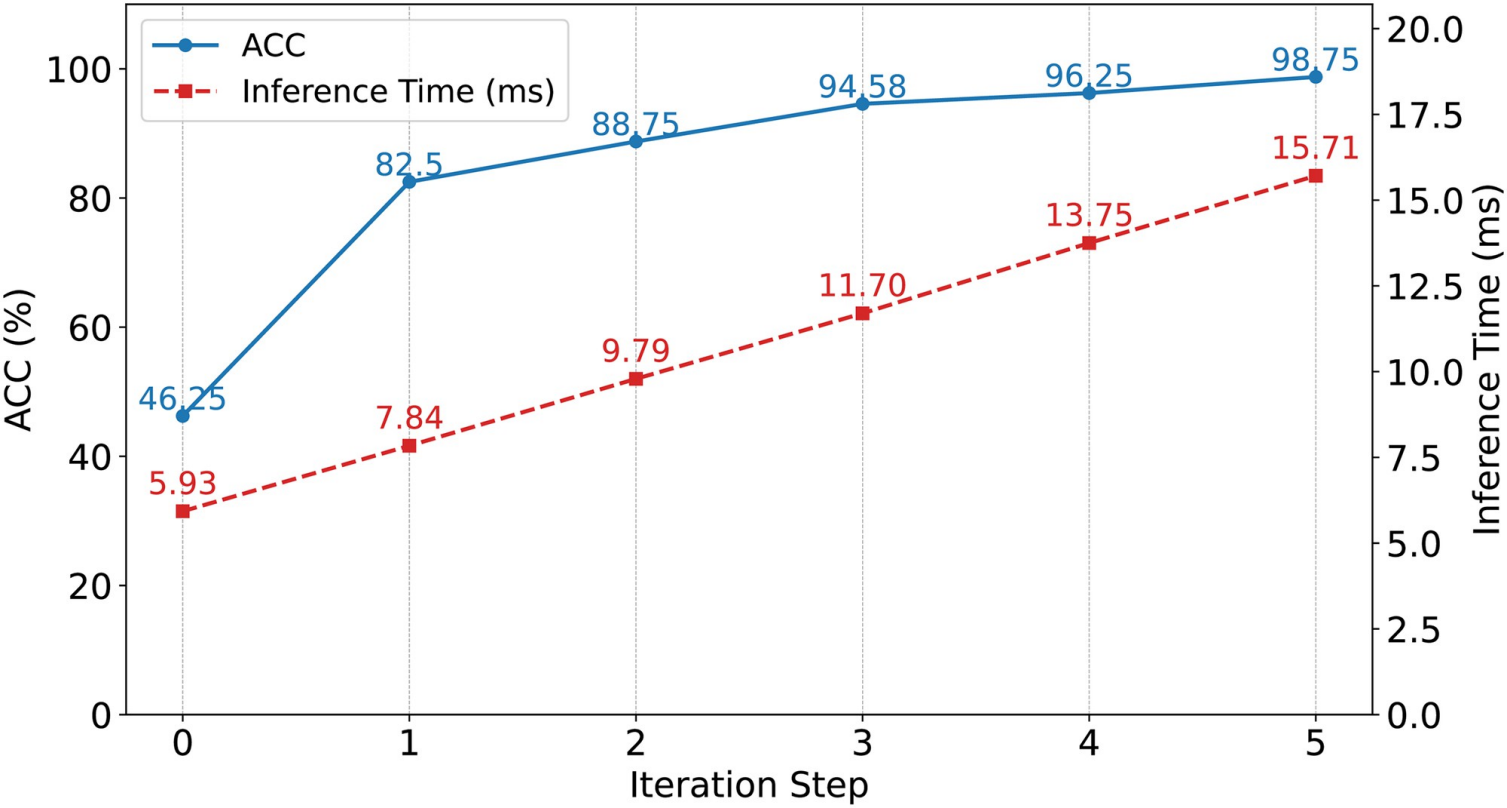
Accuracy and inference time across iteration steps.

**Group C**: As illustrated in Fig 6, the model’s accuracy continuously improved with an increasing number of iterative refinement cycles. This enhancement was attributed to each iteration’s ability to more effectively utilize the limited sample data, thereby enabling more comprehensive feature extraction. However, each iteration required an additional complete forward and backward propagation, resulting in an increase in inference time. Additionally, during the fine-tuning phase, multiple independent parameter copies needed to be maintained. Although this did not affect the main model parameters, it did lead to increased memory consumption and computational overhead. Fig 7 demonstrates that the proposed method had a parameter size of 8.70 MB and a total memory footprint of 21.75 MB, which was slightly higher than that of other common methods. Nevertheless, in a few-shot environment, the accuracy of the proposed method significantly outperformed that of the other methods. Specifically, iterative refinement increased the computation time per iteration by approximately 1.9 ms, resulting in a total inference time of 15.71 ms. However, the accuracy improved by 52.5%. Although the parameter size increased by several times compared to other methods, the accuracy correspondingly increased by approximately 30%, reaching 98.75%. This additional computational cost was justified by achieving more effective detection under few-shot conditions, thereby offering greater advantages in resource-constrained real-world applications. Moreover, the increases in time and parameter size were not substantial and remained within acceptable limits. Therefore, the proposed method maintained high practicality and efficiency while enhancing detection performance.

From three groups of experiments, we can draw three conclusions:

(1) Our method converges quickly, allowing for the selection of fewer training rounds to maintain high accuracy and detection rates while improving model efficiency.
(2) In few-shot scenarios, this method performs excellently on both CICIDS2017 and CICIDS2018 datasets and demonstrates outstanding generalization ability.
(3) Our method strikes a balance between computational overhead and accuracy, ensuring that the modest increases in inference time and memory usage are appropriately offset by substantial improvements in detection performance.

## 5 Discussion

### 5.1 Comparison with related work

The proposed few-shot network intrusion detection method based on self-attention can swiftly adapt to new tasks. It can handle various network situations and is suitable for different access methods, thereby offering significant practical value. Given the lack of extensive applications of few-shot network intrusion detection methods, relatively few options are available for comparison.

In this study, we conducted a comparative analysis of our method and recent few-shot network intrusion detection methods to validate the exceptional performance of our proposed method. Table 6 provides a comparison with other research findings encompassing the datasets used, sample sizes, accuracy of the experimental results, and detection rates. These two metrics were used to evaluate the efficacy of the proposed method.

**Table 6:** Comparison of detection results and the number of samples in the proposed method and related research works.

Method	Dataset	Samples	ACC (%)
Meta-learning (2022) [44]	CICIDS2017	5	97.43
Meta-learning (2022) [44]	CICIDS2017	10	97.56
FS-IDS (2022) [45]	CICIDS2017	5	97.51
SPN (2023) [46]	CICIDS2017	5	94.73
GDE (2023) [36]	CICIDS2018	140	99.13
MAML with L2F (2023) [47]	CICIDS2017	10	94.66
MAML with L2F (2023) [47]	CICIDS2018	5	96.24
MAML with L2F (2023) [47]	CICIDS2018	10	97.92
Siamese Network (2023) [48]	CICIDS2017	1	80.81
MAML+CNN (2023) [9]	FSIDS-IOT	5	89.64
MetaMRE (2023) [49]	CICIDS2017	5	91.47
MetaMRE (2023) [49]	CICIDS2017	10	93.84
FE-MTDM (2023) [50]	CICIDS2017	65341	99.17
Graph2vec+RF (2023) [51]	CICIDS2018	9936	99.76
FML (2024) [52]	CICIDS2017	10	87.27
Res-Natural GAN (2024) [38]	CICIDS2018	5	94.47
Res-Natural GAN (2024) [38]	CICIDS2018	10	95.52
Res-Natural GAN (2024) [38]	CICIDS2018	15	95.57
**Proposed method**	CICIDS2017	5	99.18
**Proposed method**	CICIDS2017	10	99.69
**Proposed method**	CICIDS2018	5	96.91
**Proposed method**	CICIDS2018	10	98.75

We summarized and compared recent studies utilizing the CICIDS2017 and CICIDS2018 dataset using ACC and DR as evaluation metrics. It is worth noting a continual few-shot learning method using meta-learning for intrusion detection [44] and a few-shot network intrusion detection method based on MAML with L2F [47], both of which are based on meta-learning approaches. The experimental results presented in Table 6 highlight the impressive performance of the proposed method. Notably, FE-MTDM [50] utilized just 1% of the CICIDS2017 dataset, while GDE [36], FE-MTDM [50], and Graph2vec+RF [51] represent the total number of samples used for training. In contrast, the sample sizes for the other methods reflect the number of training samples per class. Additionally, FSIDS-IOT [9] is a hybrid dataset that comprises a mix of five data types drawn from both the CICIDS2017 and CICIDS2018 datasets.

The ACC for the CICIDS2017 dataset was as high as 99.69%, whereas the ACC for the CICIDS2018 dataset reached a maximum of 98.75%. Compared with a few-shot network intrusion detection method based on MAML with L2F (97.92%) and a method with Res-Natural GAN (95.52%), our method exhibited improvements of 0.83% and 3.23%, respectively. Although methods for early and accurate network intrusion detection using graph-embedding technology perform better, they require a significant number of samples for support. By contrast, our method effectively reduces the number of training tasks and can adeptly learn new knowledge from a small number of samples, aiding in the simultaneous detection of previous attack types. Consequently, it achieves higher accuracy and detection rates.

### 5.2 Minimal few-shot

From Tables 4 and 5, it can be observed that in the CICIDS2017 dataset, as the number of samples increased from 1 to 5, all performance metrics of the model exhibited a significant upward trend. This indicates that the model maintained good robustness and stability even with a small sample size. In contrast, in the CICIDS2018 dataset, when the number of samples was 3, the detection rate and specificity were slightly lower than when the number of samples was 1. However, upon increasing the number of samples to 5, the performance metrics showed significant improvement. This fluctuation may be attributed to the inherent complexity of the dataset or the model’s insufficient generalization ability under extremely small sample sizes.

These results also highlight the stability issues of the proposed method under extremely small sample conditions (k = 1, 3), as certain classes within the dataset were difficult to adequately learn with limited samples, resulting in decreased performance when the number of samples increased. In this regard, we discuss the following: Compared to CICIDS2017, CICIDS2018 may encompass more diverse and complex attack types, making it challenging for the model to capture all class features under small sample conditions. Although the self-attention mechanism and iterative refinement strategy performed excellently when k = 5 and k = 10, the task became considerably more difficult under extremely small sample conditions (k = 1, 3). Therefore, the model may require further regularization or data augmentation techniques to enhance its generalization capability.

### 5.3 Position embedding

Although this study employed the classical sinusoidal positional encoding, demonstrating advantages in model simplicity and training efficiency, this approach still has certain limitations. Firstly, sinusoidal positional encoding generates positional representations based on fixed functional forms, lacking flexibility tailored to specific application scenarios. When handling tasks such as network traffic analysis, which exhibit highly dynamic and unstable characteristics, fixed positional encoding may fail to effectively capture the complex variation patterns of the traffic, resulting in suboptimal model performance in response to different traffic dynamics. Additionally, sinusoidal positional encoding cannot dynamically adjust positional representations in real-time based on traffic changes, limiting the model’s adaptability in real-time traffic prediction and anomaly detection. Given that network traffic often displays bursty and nonlinear features, the fixed sinusoidal encoding struggles to adequately reflect these variations, potentially affecting the model’s prediction accuracy and robustness. Therefore, future research could explore the development of more adaptive positional encoding methods optimized for dynamic traffic characteristics, thereby enhancing the model’s performance in complex and fluctuating network environments.

## 6 Limitations

### 6.1 Data imbalance

In practical applications, class distribution imbalance or skewness is a common and challenging issue. Our study primarily focused on detection with a limited number of samples and did not specifically address the problem of data imbalance. However, to some extent, our method was able to extract more discriminative features under constrained data conditions, thereby enhancing the model’s detection performance and mitigating the effects of class distribution imbalance [53]. Nevertheless, it remained unable to fully resolve issues related to class imbalance or skewness. To overcome this limitation, future research could consider incorporating more advanced balancing techniques, such as data augmentation, resampling strategies, or specially designed loss functions, to enhance the model’s robustness and generalization capabilities in imbalanced class environments while maintaining high model efficiency.

### 6.2 Real-time processing

The self-attention mechanism employed in this study demonstrated excellent performance in capturing sequential dependencies. However, its high computational complexity posed significant challenges for large-scale real-time processing. In future work, we plan to explore lightweight self-attention models and distributed computing methods to improve the model’s computational efficiency and processing speed without significantly compromising detection performance. This would enable the efficient handling of large-scale real-time data, making the model more applicable to real-world scenarios that require rapid and scalable processing.

### 6.3 Scalability

The iterative refinement strategy utilized in this research may encounter substantial scalability challenges as task complexity increases or the number of classes grows. This issue is not unique to our method. In fact, most existing algorithms face difficulties under similar circumstances. Consequently, scalability research has become a crucial direction, particularly for fine-grained classification, to alleviate scalability concerns. Future work should focus on developing more efficient optimization algorithms and fine-grained classification methods to enhance the scalability of iterative refinement strategies in complex tasks. This would provide new insights and solutions for addressing scalability problems, thereby improving the applicability and effectiveness of the proposed method in more diverse and demanding environments.

## 7 Conclusion

This paper presents an elevated few-shot network intrusion detection method via self-attention mechanisms and iterative refinement. The proposed method is a deep neural network model consisting of embedding, encoding, and classification layers. By leveraging the self-attention mechanism, this method extracts extensive global information in spatial sequences, thereby enabling the extraction of more effective network traffic features for few-shot network intrusion detection. The proposed method was evaluated using the CICIDS2017 and CICIDS2018 datasets. The experimental results demonstrate that our method achieves detection rates of 99.90% and 98.23% under 10-sample conditions, indicating its ability to maintain high accuracy and detection rates even with extremely limited samples. In future work, we will develop more comprehensive and diverse few-shot datasets and detection models, focusing on noisy samples and adversarial attacks in real-world scenarios. Advanced data augmentation techniques will be employed, and adversarial training methods will be thoroughly explored to comprehensively evaluate and enhance the model’s robustness and effectiveness in complex environments. Additionally, the model’s generalizability will be further validated in actual network environments to ensure its stable performance and broad applicability in real-world application settings.
